# Multiscale simulations that incorporate patient-specific neural network models of platelet calcium signaling predict diverse thrombotic outcomes under flow

**DOI:** 10.1371/journal.pcbi.1013085

**Published:** 2025-05-06

**Authors:** Kaushik N. Shankar, Talid Sinno, Scott L. Diamond

**Affiliations:** Department of Chemical and Biomolecular Engineering, Institute for Medicine and Engineering, University of Pennsylvania, Philadelphia, Pennsylvania, United States of America; Clemson University, UNITED STATES OF AMERICA

## Abstract

During thrombosis, platelets rapidly deposit and activate on the vessel wall, driving conditions such as myocardial infarction and stroke. The complexity of thrombus formation in pathological flow geometries, along with patient-specific pharmacological responses, presents an opportunity for computational modeling to help deliver novel diagnostic and therapeutic insights. In the present study, we employed a multiscale 3D computational model that incorporates unique donor-derived neural networks (NNs) trained with platelet calcium mobilization traces under combinatorial exposure to 6 agonists (n = 10 donors). The 3D model comprises four modules: a donor-specific NN model for platelet calcium mobilization, a lattice kinetic Monte Carlo solver for tracking platelet motion and bonding, a finite volume method solver for modeling soluble agonist release and convective-diffusive transport, and a lattice Boltzmann method solver for predicting the blood velocity field. Simulations were conducted for platelets from individual blood donors under venous and arterial flow conditions on a defined collagen surface, examining the effects of inhibiting ADP and TXA_2_, as well as the influence of nitric oxide and prostacyclin. The results reveal significant individual variability in platelet responses, influencing simulated thrombus growth dynamics and emphasizing the importance of personalized models for predicting thrombotic behavior. This approach enables consideration of patient-specific platelet signaling, drug responses, and vascular geometry for predicting thrombotic episodes, essential for advancing precision medicine and improving patient outcomes in thrombotic conditions.

## Author summary

Blood clots play a key role in serious conditions such as heart attacks and strokes. In this work, we utilized a computer model to understand how platelets, the key cellular components in blood clot formation, behave in different individuals. The model uses data from 10 real individuals to simulate how platelets respond to blood clotting triggers, as well as drugs that reduce or prevent clot formation and growth. By examining the flow of blood and the behavior of platelets, we were able to predict how clots might form in different scenarios. Importantly, our results show that individual responses to clotting triggers and medications vary, which means that treatments may need to be tailored to each individual. This research moves us closer to personalized approaches in preventing and treating blood clot-related diseases, potentially improving patient outcomes.

## Introduction

Thrombosis, the formation of blood clots within blood vessels, remains a critical area of study due to its implications in various medical conditions such as myocardial infractions and strokes. The complexity of thrombus formation, influenced by both biochemical and mechanical factors, necessitates advanced computational modeling to improve our understanding and guide clinical interventions. Over the past two decades, significant strides have been made in developing these models, yet challenges remain in accurately predicting thrombus behavior under patient-specific physiological and pathological conditions.

Models for thrombosis may be classified into continuum, discrete particle, and hybrid multiscale methods [1,2]. Continuum models treat blood and its components as continuous media, using partial differential equations to describe the flow and biochemical reactions involved in clot formation [3–6]. These models excel at capturing large-scale behaviors but may lack the ability to fully resolve individual cellular interactions. On the other hand, discrete particle models represent blood cells and platelets as individual entities, and naturally resolve the detailed (stochastic) interactions between them [7–10]. While they offer high-resolution insights into cellular dynamics, they are generally computationally intensive and challenging to employ at the vessel scale over the minutes-timescale relevant to thrombus growth. Multiscale models integrate both approaches, combining continuum descriptions of blood flow with discrete representations of cellular interactions to provide a comprehensive framework for simulating thrombus formation [11–17]. These models achieve a balance between detailed representation and computational efficiency.

In prior work, we developed a 2D multiscale model for thrombus growth under flow, utilizing a neural network model for platelet calcium signaling and activation [18], a lattice kinetic Monte Carlo module to track platelet motion and deposition on a growing clot mass under flow [11,19,20], a finite volume method solver for computing agonist species concentration fields (specifically ADP and TXA_2_) described by a convection-diffusion-reaction equation, and a lattice Boltzmann method solver for tracking and updating the fluid velocity field as the clot grows [11,13]. Subsequent research expanded this model into a fully 3D version using a combination of open-source libraries and in-house codes, which we parallelized to handle complex simulations efficiently [14,21]. This iterative development has allowed us to incorporate simulations of thrombus formation and growth under a variety of physiologically relevant 3D domains, such as cylindrical blood vessels and stenoses.

The present work employs this model to study how variations in platelet signaling between individuals impact the dynamics of thrombus growth. Platelets exhibit considerable variability in receptor expression and functional responses to agonist stimulation from individual to individual, which can significantly influence thrombus formation and stability [22–24]. The multiscale approach enables the seamless integration of individual-specific models for platelet signaling and activation without requiring changes to its other components. The model incorporates detailed representations of platelet calcium signaling and integrin activation, using neural networks trained for individual blood donors by Lee and Diamond [25]. We explore individual-specific platelet signaling by inhibiting various agonist signaling pathways, examining their impact on the dynamics of thrombus growth under two distinct conditions: (1) venous shear rates in a cylindrical vessel and (2) arterial shear rates in a stenotic geometry. The simulations reveal significant individual differences in platelet responses to agonist stimulation under both conditions, even within a healthy cohort. This variability impacts the dynamics of thrombus formation explored under several scenarios involving pharmacological modulation. The personalized simulation approach presented in this paper addresses a significant gap in existing modeling efforts, which often overlook the variability in platelet function among different individuals. Incorporating individual platelet phenotypes into the multiscale model presents an important direction forward for the development of targeted anti-thrombotic therapies and the broader field of personalized medicine.

## Methods

**Fig 1:**
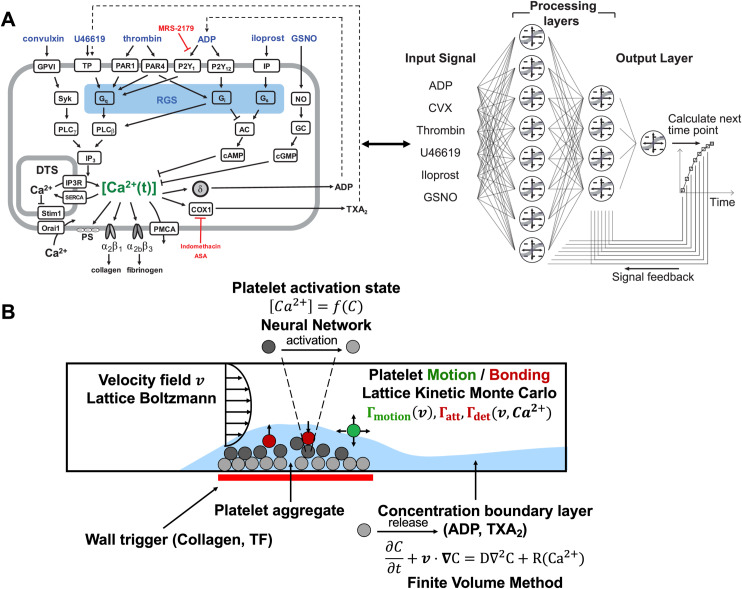
Multiscale model of platelet activation in response to combinatorial agonist stimulation and thrombus growth under flow. (A) The six agonists used in this study (ADP, convulxin, thrombin, U46619, iloprost, GSNO) and their respective platelet signaling pathways, all of which converge upon intracellular calcium mobilization. A two-layer, 12-node neural network architecture was employed to predict donor-specific platelet calcium mobilization. Agonist concentrations at a given time point were fed into the processing layers; the layers then integrated the input signal with feedback at t = 1, 2, 4, 8, 16, 32, 64 and 128 seconds to calculate [Ca^2+^]_i_ at the next time point. (B) The multiscale simulation of thrombus growth under flow required simultaneous solutions of the instantaneous velocity field over a complex and evolving platelet boundary by LB, concentration fields of ADP and TXA_2_ by FVM, individual intracellular platelet state ([Ca^2+^]_i_) and release reactions (R) for ADP and TXA_2_ by NN, and all platelet positions and adhesion/detachment by LKMC.

The major components of the multiscale framework used in this study are shown schematically in Fig 1; four distinct modeling elements are combined to capture a variety of physical, biological, and chemical processes that contribute to thrombus growth and evolution. At the heart of the framework is a model for evolving platelet positions and adhesion states using the lattice kinetic Monte Carlo (LKMC) algorithm. The LKMC domain is discretized into a cubic lattice where each lattice point represents an allowable position for platelets. Platelets that are adjacent to other platelets or other surfaces have an opportunity to bind, or if already bound, detach. A rate database of all possible events, including platelet motion, adhesion, and detachment, is constructed initially and updated after each event has been executed. Events are executed sequentially in an order that is biased by their rates [26]. Rates for platelet motion are established by a combination of diffusion and the local blood flow velocity profile. Adhesion and detachment rates are functions of platelet activation and the local shear rate, capturing the dynamic interaction between platelets and their environment (see below). These functions take into consideration the extent of inside-out platelet signaling, the shear-dependent breaking of ligand-receptor bonds, as well as the impact of von Willebrand factor (vWF) mediating enhanced platelet adhesion at pathological shear rates [14].

The blood velocity field in the domain is updated quasi-statically as the platelet aggregate grows using the Lattice Boltzmann (LB) method [27]. Blood is modeled as an incompressible Newtonian fluid governed by the Navier-Stokes equations, which in the LB method are solved by executing a sequence of collisional and streaming operations on fictive fluid particles. Red blood cells are not explicitly resolved but their effects on platelet transport and margination are included by imposing a non-uniform excess near-wall platelet concentration at the inlet of the simulation domain [28], where platelets are inserted with a rate determined by their mixing cup inlet concentration, with the bulk platelet concentration set at 1.5 × 10^5^ μL^-1^ [14]. We note that while platelets may not be effectively pushed back toward the vessel wall after passing a stenotic region due to the absence of explicitly resolved red blood cells, it is assumed that its impact on thrombus formation is minimal, as platelet aggregation predominantly occurs at the apex of the stenosis where shear-induced vWF-mediated adhesion plays a dominant role (see Results). Next, the spatiotemporal concentrations of soluble agonists – restricted to ADP and TXA_2_ in the present study – are modeled by coupled convection-diffusion-reaction (CDR) partial differential equations. In addition to diffusion and advection via flow, agonists are produced via dense granule release or synthesis by sufficiently activated platelets [29]. This release is modeled using spatiotemporal sources for ADP and TXA_2_ at locations in the simulations domain where aggregated platelets became sufficiently activated beyond a critical threshold [14]. The CDR partial differential equations are solved numerically using the Finite Volume Method (FVM). Although the blood flow profile could have also been solved using FVM, handling growing platelet deposits and applying no-slip boundary conditions on a moving domain is challenging. The LB method was chosen for its ease of use and because its lattice is commensurate with the lattice used in the LKMC model, allowing for an efficient implementation where a bound platelet occupying LB nodes results in the collision operators being modified to provide a no-slip boundary condition.

In the present study, the preceding model components are assumed to be universal, i.e., they do not explicitly include individual-specific characteristics. The latter are introduced in the module that describes platelet signaling. During clot formation, platelet signaling involves combinatorial stimulation by collagen, ADP, thromboxane (TXA_2_), and thrombin, while being modulated by nitric oxide and prostacyclin from the endothelium. Platelet signal transduction triggers calcium mobilization, which initiates inside-out signaling [30]. This process includes integrin activation, the release of α and dense granules, and the synthesis of TXA_2_. In previous work by Lee and Diamond, 10 donor-specific Neural Networks (NN) were trained on Pairwise Agonist Scanning (PAS) data from 10 individual healthy blood donors (5 females, 5 males) under 40 years of age, representative of a healthy adult population [25]. Future studies involving blood phenotypes from specific patient cohorts can be contrasted to the healthy cohort in the present work. PAS data consists of time-resolved calcium responses to individual and pairwise combinations of six agonists at different concentrations [18,25]. Each trained NN then predicts donor-specific, intra-platelet calcium mobilization upon exposure to multiple biochemical stimuli, including ADP, TXA_2_, collagen, thrombin, nitric oxide donor GSNO, and prostacyclin analog iloprost. The effect of collagen and TXA_2_ are accounted for by considering equivalent effective concentrations of GPVI activator convulxin (10 nM) and TP activator U46619 (15 × [TXA_2_]) respectively [25]. The predicted time-dependent intracellular calcium concentration for any platelet*,* ${\left[Ca^{2+}\right]}_{i}(t)$, is based on the feedback vector (containing calcium concentrations from 1, 2, 4, 8, 16, 32, 64, and 128 seconds prior to the current time) and the current concentration input of the six agonists (see Fig 1A). The time-dependent platelet activation state for platelet i, $\xi_{i}(t)$, is derived by integrating the calcium concentration over time above a basal level, i.e.,


$$\xi_{i}(t)=\int {(\left[Ca^{2+}\right]}_{i}-{\left[Ca^{2+}\right]}_{0})dt.\;$$

The extent of integrin activation and inside-out signaling is estimated using a Hill function, which facilitates the determination of platelet adhesiveness, F(ξ), such that


$$F(\xi )=\alpha_{\min}+{(\alpha }_{\max}-\alpha_{\min})\frac{\xi^{n}}{\xi^{n}+\xi_{50}^{n}},$$

where n controls the sharpness of the response, and $\xi_{50}$ is the critical value for 50% activation.

By coupling the four modules together, the multiscale framework provides a robust tool for simulating thrombus growth, accommodating individual variability in platelet signaling and capturing the complex dynamics of thrombus formation under varying flow conditions. Comprehensive descriptions of each module, their methodologies, and implementation are provided in previous work [14,21]. To enable computationally efficient simulations in a reasonable amount of time, the computational framework has been parallelized to ensure balanced computational load across multiple CPUs [21]. The LB and FVM modules are implemented using the parallel open-source libraries Palabos and OpenFOAM, respectively [31,32]. In-house C++ codes based on the message passing interface (MPI) were employed for the NN and LKMC modules. To facilitate efficient information exchange between the modules, the Multiscale Universal Interface (MUI) was employed. MUI is a C++ library that makes use of non-blocking MPI messages to achieve data transfer, ensuring minimal modifications to the individual module codes [33].

## Results

**Fig 2:**
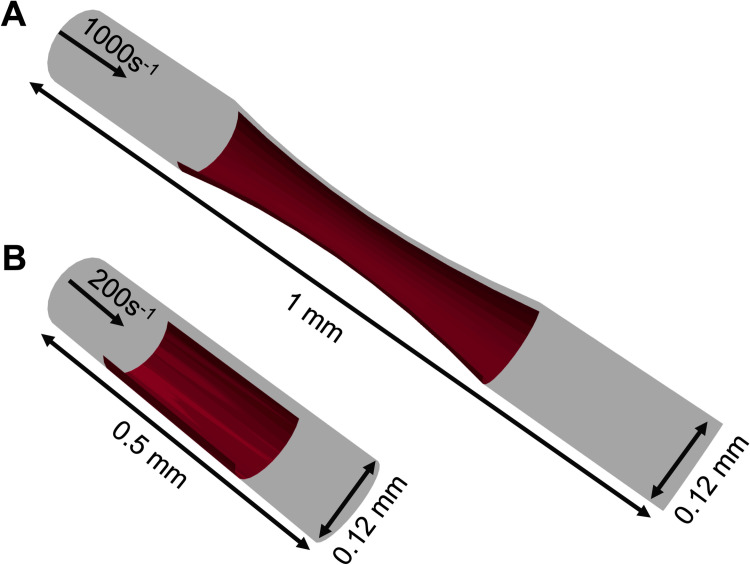
Simulation domains for donor-specific thrombus growth. (A) Stenotic arterial flow condition and (B) cylindrical venous flow condition.

The results from the donor-specific simulations of thrombus growth for 10 individual donors are presented through two case studies. In the first case, we examined a 1 mm-long stenotic vessel with an inlet diameter of 0.12 mm. Within the central 0.5 mm of this vessel, a narrowing was introduced, reducing the flow area by 75%, as depicted in Fig 2A. This stenotic area was assumed to express collagen covering up to half of the vessel’s circumference, representing the injury site triggering platelet aggregation. At the inlet, a constant wall shear rate of 1000 s^-1^, typical of arterial flow, was maintained [34]. In the second case, we explored a cylindrical domain illustrated in Fig 2B. Here, the simulation domain measured 0.5 mm in length and 0.12 mm in diameter. A semicylindrical reactive collagen surface patch with a diameter of 0.12 mm and a length of 0.25 mm, representing an injury, was placed at the center of the domain. In this case, a constant wall shear rate of 200 s^-1^, characteristic of venous flow, was maintained at the inlet [34]. For both cases, the multiscale framework was employed to simulate platelet aggregation under flow conditions over a period of 6 minutes.

### Donor-specific simulations of thrombus growth in response to antiplatelet treatment

**Fig 3:**
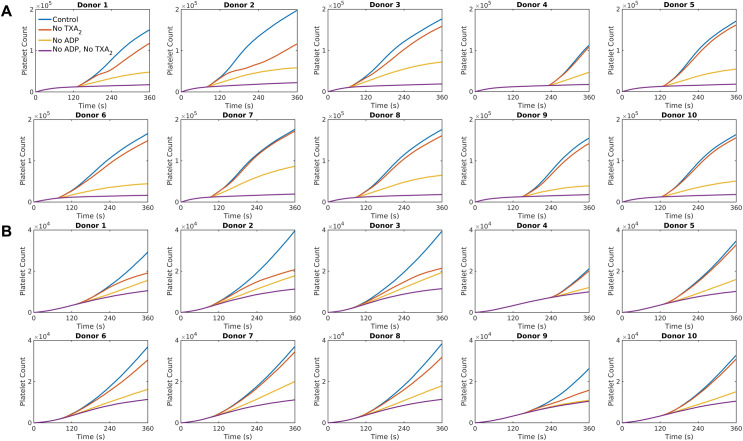
Donor-specific thrombus growth dynamics observed under different agonist conditions. Deposited platelet count predicted by the simulations for each donor with no TXA_2_, no ADP, or both, under (A) stenotic arterial flow and (B) cylindrical venous flow.

The dynamics of platelet deposition over time were studied for each donor under pharmacological modulation of platelet function by antiplatelet drugs. We first considered a control condition, where platelet deposition occurred on collagen without any inhibition of agonists. We then compared the control condition with conditions where the release of TXA_2_, ADP, or both were inhibited. The inhibition of agonists ADP and TXA_2_ was mimicked by setting their concentrations to zero in the donor-specific NNs. These settings simulate the action of antiplatelet drugs such as clopidogrel, which binds to ADP receptor P_2_Y_12_, and aspirin, which inhibits TXA₂ synthesis. These drugs are commonly used to manage thrombotic diseases, making their inclusion in the model relevant for assessing the impact of therapeutic interventions on platelet aggregation. The number of aggregated platelets as a function of time for each condition for the two case studies is presented in Fig 3. For each donor, the order of agonist potency is the same: inhibiting both ADP and TXA_2_ has the strongest effect, followed by inhibiting ADP, and TXA_2_ inhibition showing the least impact compared with the control condition. These overall trends notwithstanding, the number of aggregated platelets and the rate at which platelet deposits grow over time vary significantly among donors as shown in Fig 3. Notably, the simulations predict differing levels of sensitivity to agonist inhibition among the donors. The reduction in platelet aggregate count under TXA_2_ inhibition compared to the control condition ranges from ~30–40% for Donor 2 to <10% for Donors 4, 5, 7, and 10 for both case studies. Similarly, inhibiting ADP leads to a reduction in deposited platelet count ranging from ~40–70% compared to the control condition.

**Fig 4:**
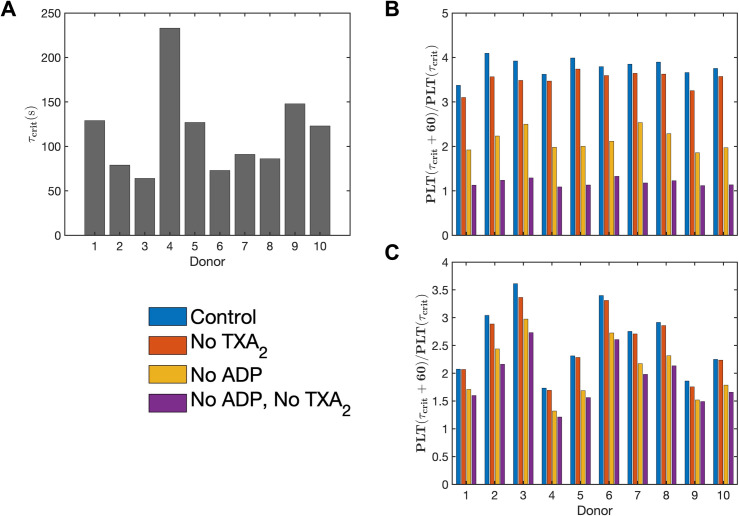
Variations in donor-specific platelet responses. (A) Time taken to reach critical platelet activation for dense granule release, $\tau_{crit}$, for each donor under collagen stimulation. Thrombus growth rate measured as the fold increase in the number of platelets deposited 1 minute after $\tau_{crit}$ for (B) stenotic arterial flow and (C) cylindrical venous flow.

**Fig 5:**
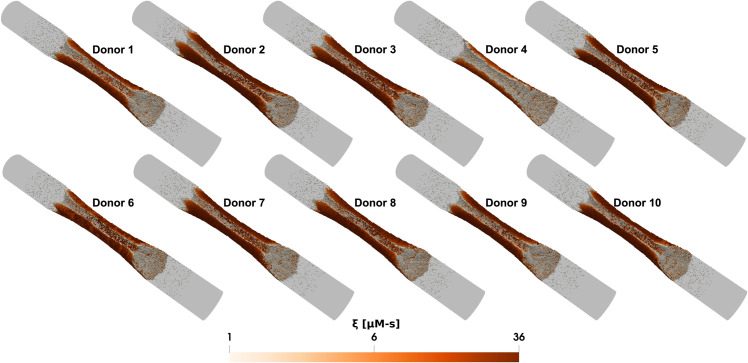
Donor-specific simulations of thrombus growth in stenotic arterial flow. Snapshots of the platelet aggregate under the stenotic arterial flow case for different donors after 6 minutes. In all subplots, white indicates inactivated platelets and red indicates highly activated platelets formed on the collagen surface.

**Fig 6:**
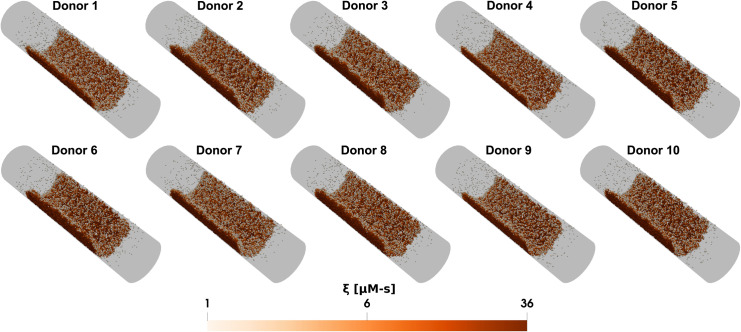
Donor-specific simulations of thrombus growth in cylindrical venous flow. Snapshots of the platelet aggregate under the cylindrical venous flow case for different donors after 6 minutes. In all subplots, white indicates inactivated platelets and red indicates highly activated platelets formed on the collagen surface.

In the model, platelets release ADP and TXA_2_ only after reaching a certain activation threshold, $\xi_{crit}$. Without other activating factors, the time required for the first layer of deposited platelets to become sufficiently activated by collagen to release dense granules varied widely among donors, ranging from about ~60 seconds for Donor 3 to ~240 seconds for Donor 4, as shown in Fig 4A. Additionally, the ability of the first monolayers of activated platelets to recruit more platelets to the thrombus (measured by the ratio of platelet counts 1 minute after dense granule release to the platelet count at the time of release) differs among donors under each condition (see Fig 4B). This variation influences both the total number of deposited platelets and their activation levels across the donor group, with the highest response (Donor 2) being ~2× that of the lowest response (Donor 4). Figs 5 and 6 provide snapshots of the deposited platelet mass for each donor after 6 minutes under the control condition for the two cases, clearly illustrating the donor-specific differences in predicted platelet aggregate sizes and their corresponding activation levels.

### Comparison between thrombus growth under venous and arterial flow conditions

**Fig 7:**
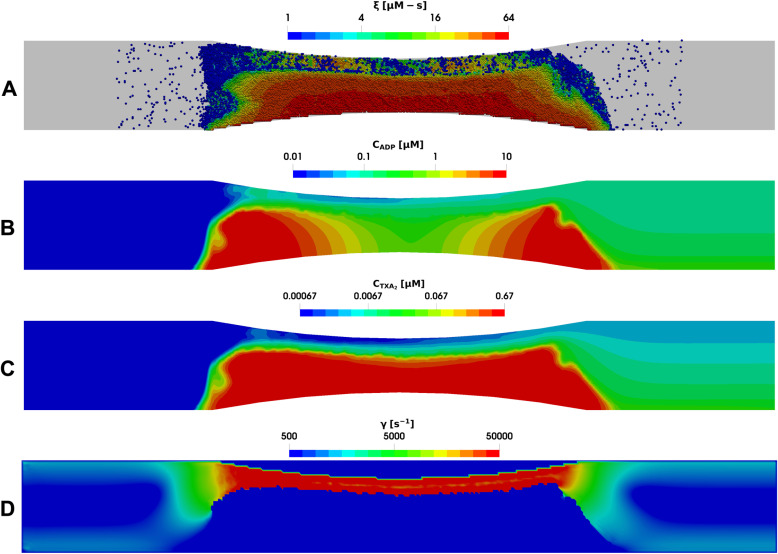
Multiscale simulation of patient-specific platelet aggregation under pathological stenotic arterial flow. (A) Platelet activation (blue indicates inactivated and red, highly activated) and deposition in the presence of released (B) ADP and (C) TXA_2_, and (D) the local shear rate contours plotted along the center of the vessel after 360 seconds.

**Fig 8:**
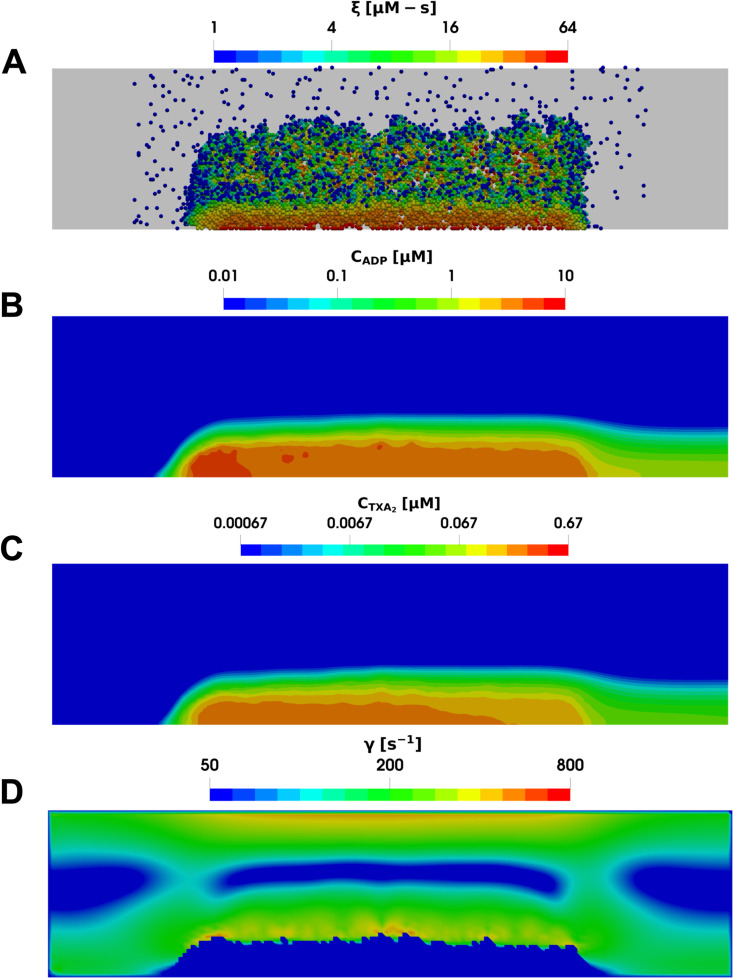
Multiscale simulation of patient-specific platelet aggregation under cylindrical venous flow. (A) Platelet activation (blue indicates inactivated and red, highly activated) and deposition in the presence of released (B) ADP and (C) TXA_2_, and (D) the local shear rate contours plotted along the center of the vessel after 360 seconds.

The dynamics of platelet aggregation over time follow a similar trend in both venous and arterial flow cases, as shown already in Fig 3. However, the preferred location of platelet aggregation within the domain differs significantly between the two cases. Under venous flow conditions, platelets tend to aggregate towards the upstream end of the collagen surface. In contrast, in the stenotic case platelets aggregate in areas with higher local shear rates. Figs 7 and 8 illustrate this difference with snapshots for Donor 2, showing the aggregated platelets, their activation levels, the spatial concentration distribution of ADP and TXA_2_, and the local shear rate around the clot along a central slice of the domain after 6 minutes for both arterial and venous cases. This difference is due to the action of von Willebrand factor, which changes from a globular conformation at low shear rates to a stretched one at shear rates greater than 3000–8000 s^-1^, thereby mediating enhanced platelet aggregation [35,36]. Additionally, the morphologies of the clots differ significantly between the venous and arterial cases. Platelet aggregates are more tightly packed under arterial flow conditions and exhibit higher porosity under venous conditions. As a result, intra-clot agonist concentrations are about an order-of-magnitude higher in the stenotic arterial case as compared to the cylindrical venous case. The higher agonist concentrations observed in the stenotic arterial case could have implications such as increased risk of arterial blockages in stenotic vessels, emphasizing the need for targeted therapeutic interventions to manage arterial thrombosis.

### Donor-specific simulations of thrombus growth in response to endogenous platelet antagonists

**Fig 9:**
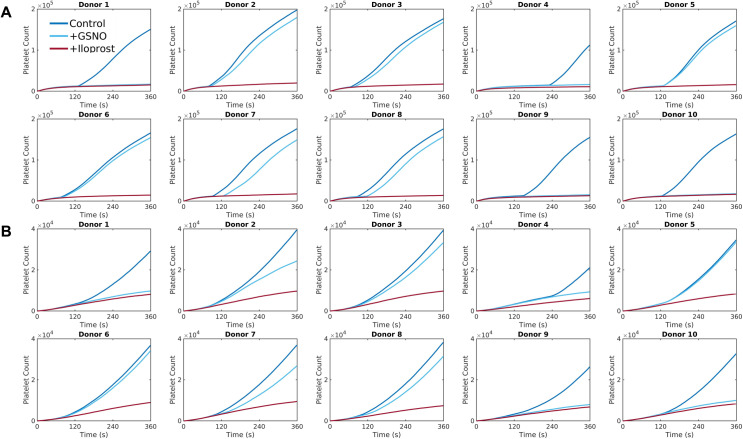
Donor-specific thrombus growth dynamics observed under antagonist stimulation. Deposited platelet count predicted by the simulations for each donor with GSNO and iloprost stimulation under (A) stenotic arterial flow and (B) cylindrical venous flow.

The impact of endothelium-derived platelet antagonists, nitric oxide and prostacyclin, was also assessed for each donor in the cohort. During NN training, GSNO and iloprost were used as analogs for nitric oxide and prostacyclin, respectively. The results from the multiscale simulations under GSNO and iloprost stimulation for both stenotic arterial flow conditions and venous flow conditions in a cylindrical vessel are presented in Fig 9. The simulations reveal a consistent order of potency among different donors: iloprost demonstrates the strongest inhibitory effect, followed by GSNO, when compared to the control condition. Notably, the responses to GSNO varies significantly among donors. Donors with a strong collagen response (low $\tau_{crit}$) exhibit minimal inhibition compared to the control condition. Conversely, 4 donors with a weaker collagen response (donors 1, 4, 9, and 10) show potent inhibition under GSNO stimulation, resulting in the absence of dense granule release from the first monolayer of deposited platelets. However, Donor 5, despite exhibiting a weaker collagen response, does not show a similar inhibitory response. GSNO appears to have a very weak inhibitory effect on Donor 5, despite having similar values of $\tau_{crit}$ when compared to Donor 10. This difference may be due to variations in platelet phenotypes, where nitric oxide signaling could interact differently with platelet signaling pathways in these donors. Iloprost, on the other hand, is a highly potent inhibitor of collagen-induced platelet activation across all donors. In the presence of iloprost, dense granule release does not occur for any donor, demonstrating its robust efficacy in modulating platelet activation.

### Donor-specific simulations of thrombus growth in response to the inclusion of coagulation via wall-derived tissue factor

**Fig 10:**
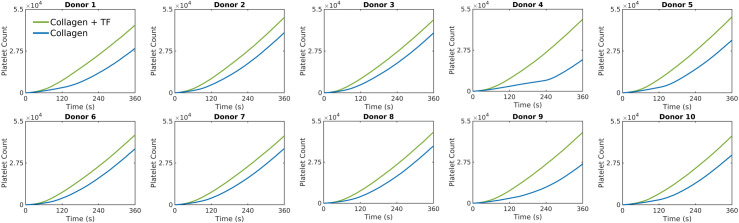
Donor-specific thrombus growth dynamics observed in the presence of wall-derived TF. Deposited platelet count predicted by the simulations for each donor with and without TF under cylindrical venous flow.

In addition to collagen, wall-derived tissue factor (TF) cn enhance platelet aggregation via thrombin production through the coagulation cascade, particularly under venous flow conditions [37]. Therefore, we have explored the donor-specific effects of clotting in the presence and absence of wall-derived TF for the venous cylindrical case. To model intra-clot thrombin concentration, we utilized a reduced ODE model for thrombin generation under venous flow developed by Chen and Diamond [38]. The reduced model is guided by the observation that most thrombin is sequestered within the fibrin-rich clot core [39]. Although the ODE model neglects spatial gradients, which is a limitation, it still captures the key thrombin dynamics during venous thrombus formation [38]. This model was used to prescribe the concentration of thrombin within the clot, which is an input to the NN model for platelet calcium signaling. The dynamics of observed platelet count over time for each donor are presented in Fig 10. Platelet deposition on collagen + TF (1 molecule/ $\mu m^{2}$) was ~ 20–100% higher compared to collagen alone, with the most significant differences observed in donors with weaker collagen responses (high $\tau_{crit}$).

## Discussion

In this study, we investigated donor-specific thrombus growth simulations using neural network models of platelet signaling tailored to individual donors. We conducted simulations in two distinct geometries: a stenotic vessel and a cylindrical vessel. The stenotic vessel simulates arterial conditions with a high wall shear rate of 1000 s^-1^. This setup is critical for understanding thrombus formation in conditions such as arteriosclerosis, where narrowed vessels are common. The cylindrical vessel, maintained at a venous shear rate of 200 s^-1^, provides insights into thrombus formation under venous flow conditions. We also examined various antiplatelet treatments by inhibiting the production of soluble agonists ADP and TXA_2_, and by stimulating platelets with antagonists such as GSNO and iloprost. The simulations demonstrated considerable individual variability in platelet responses to agonist stimulation, even among a healthy sample of individuals, which significantly affects thrombus growth dynamics. For instance, the time required for the first layer of platelets to release dense granules through collagen-mediated activation was found to vary considerably, ranging from approximately one minute to four minutes. This variability in activation thresholds and subsequent platelet aggregation highlights the need for personalized models to accurately predict thrombotic behavior. Following up on our previous studies which established the multiscale model for thrombus growth [14,21], this work demonstrates the utility of the modeling framework in capturing patient-specific variability in platelet signaling and thrombotic responses. By incorporating a neural network-based approach, this study highlights the ability to account for differences in platelet activation and aggregation among individuals, providing a more robust means to predict and understand thrombus formation.

Building on previous experimental and computational studies, our results align with well-established trends in thrombus formation. The timescale for platelet dense granule release observed in our simulations is consistent with prior work by Flamm et al and Lu et al [11,13], who developed models for platelet aggregation validated against microfluidics experiments, as well as studies by Bark et al [40], who described an initial lag time of 1–3 minutes followed by rapid platelet aggregation. Additionally, our findings on the shear-dependent nature of platelet aggregation and preferential deposition at the apex of stenotic regions at pathologically high shear rates > 3000s^-1^ are in agreement with computational models developed by Yazdani et al [12], Zhussupbekov et al [41], and Mehrabadi et al [42], which capture similar trends observed experimentally [34,43]. Furthermore, our ranking of platelet sensitivity to antiplatelet treatments is consistent with microfluidics experiments which have characterized platelet responses to pharmacological inhibition [11,13,44]. These comparisons reinforce the validity of our modeling approach and underscore its potential to capture inter-individual variability in thrombus formation.

Looking ahead, several enhancements and extensions to the current model could be pursued. Although we have accounted for differences in platelet responses to combinatorial agonist stimulation between donors, we have not accounted for intra-donor platelet heterogeneity. Platelets within an individual can exhibit varying degrees of sensitivity to agonist stimulation, leading to a spectrum of activation responses. Future enhancements to the model should incorporate this intra-donor variability, perhaps by developing subpopulations of platelet phenotypes within the neural network framework. This refinement would better capture the nuances of platelet behavior and provide a more detailed and accurate representation of thrombus formation. Additionally, in our simulations, we assumed complete inhibition of agonist production following antiplatelet treatment. However, inhibition levels can vary due to differences in drug efficacy, and future work could explore graded inhibition models to better capture these variations and assess their impact on thrombus formation dynamics. Furthermore, incorporating more detailed vascular geometries and extending the model to include dynamic feedback mechanisms between thrombus formation and systemic hemodynamics would provide a more comprehensive understanding of thrombosis in vivo [45–47]. Lastly, developing neural networks trained on a wider range of platelet phenotypes, including those from patients with different medical conditions, could enhance the model’s applicability and reliability in diverse clinical scenarios.

In conclusion, the ability to incorporate platelet phenotypes into thrombus growth models may have significant implications for personalized anti-thrombotic therapies. By understanding the specific platelet activation profiles of individual patients, tailored therapeutic interventions may be designed to more effectively prevent or manage thrombotic events. This personalized approach extends beyond anti-thrombotic therapies to encompass broader applications such as hemostasis and trauma patient outcomes [48,49]. For instance, neural networks trained rapidly in a clinical setting on platelet phenotypes from trauma patients could be used to predict and manage bleeding complications in real-time, improving patient outcomes through precision medicine. Continued advancements in model refinement, coupled with clinical validation, will pave the way for more accurate and effective personalized therapeutic strategies in the future.
